# Ultrahigh Energy and Power Density in Ni–Zn Aqueous Battery via Superoxide-Activated Three-Electron Transfer

**DOI:** 10.1007/s40820-024-01586-z

**Published:** 2024-11-29

**Authors:** Yixue Duan, Bolong Li, Kai Yang, Zheng Gong, Xuqiao Peng, Liang He, Derek Ho

**Affiliations:** 1 School of Mechanical Engineering, State Key Laboratory of Intelligent Construction and Healthy Operation and Maintenance of Deep Underground Engineering, Sichuan University, Chengdu, 610065 People’s Republic of China; 2 Department of Materials Science and Engineering, City University of Hong Kong, Kowloon, Hong Kong, 999077 People’s Republic of China; 3 Hong Kong Centre for Cerebro-Cardiovascular Health Engineering, Hong Kong Science Park, Hong Kong, 999077 People’s Republic of China; 4 School of Mechanical Engineering, Sichuan University, Chengdu, 610065 People’s Republic of China

**Keywords:** Superoxide, Multiple electron transfer, Ni aqueous battery, AIoT power source, Wearable health monitoring

## Abstract

Aqueous Ni–Zn microbatteries are safe, reliable and inexpensive but notoriously suffer from inadequate energy and power densities. Herein, we present a novel mechanism of superoxide-activated Ni substrate that realizes the redox reaction featuring three-electron transfers (Ni ↔ Ni^3+^). The superoxide activates the direct redox reaction between Ni substrate and KNiO_2_ by lowering the reaction Gibbs free energy, supported by in-situ Raman and density functional theory simulations. The prepared chronopotentiostatic superoxidation-activated Ni (CPS-Ni) electrodes exhibit an ultrahigh capacity of 3.21 mAh cm^−2^ at the current density of 5 mA cm^−2^, nearly 8 times that of traditional one-electron processes electrodes. Even under the ultrahigh 200 mA cm^−2^ current density, the CPS-Ni electrodes show 86.4% capacity retention with a Columbic efficiency of 99.2% after 10,000 cycles. The CPS-Ni||Zn microbattery achieves an exceptional energy density of 6.88 mWh cm^−2^ and power density of 339.56 mW cm^−2^. Device demonstration shows that the power source can continuously operate for more than 7 days in powering the sensing and computation intensive practical application of photoplethysmographic waveform monitoring. This work paves the way to the development of multi-electron transfer mechanisms for advanced aqueous Ni–Zn batteries with high capacity and long lifetime.

**Supplementary Information:**

The online version contains supplementary material available at 10.1007/s40820-024-01586-z.

## Highlights

- Efficient activation of Ni electrode employs chronopotentiostatic superoxidation.
- Novel superoxide activation mechanism realizes the redox reaction with three-electron transfer (Ni ↔ Ni^3+^).
- As-prepared CPS-Ni||Zn batteries exhibit simultaneously ultrahigh energy and power densities.

**Supplementary Information:**

The online version contains supplementary material available at 10.1007/s40820-024-01586-z.

## Graphical abstract

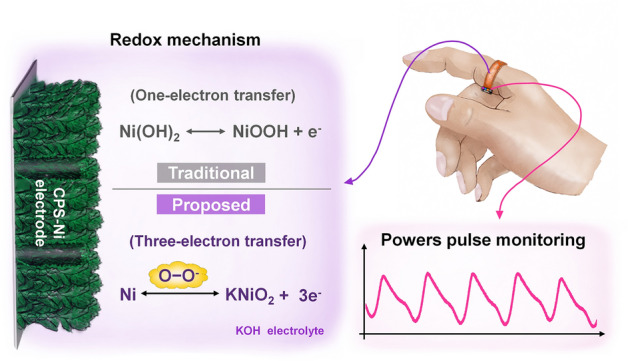

## Introduction

With the growing demand for wearable electronics as enabled by the artificial intelligence of things (AIoT) revolution, micro-power sources are facing more stringent requirements, such as simultaneously high-power output operation, high capacity, small physical size, and lightweight [1–4]. A range of miniaturized power sources (a.k.a. microbatteries) featuring a footprint of ≤ 1 cm^2^ have been developed to meet these demands [5–7]. Furthermore, they also demonstrate a stable voltage output and are capable of delivering energy under extended operation. Aqueous battery systems have attracted extensive research interest due to their natural nontoxicity and nonflammability [8, 9]. Their safe fabrication environment opens up more prospects for advanced manufacturing techniques to be applied [10]. As a classic electrochemical system with high rate capabilities, Ni||Zn batteries are characterized by a single-electron transfer between Ni(OH)_2_/NiOOH with fast ion/electron transport [11, 12]. Furthermore, the high discharge voltage plateau (≈ 1.8 V) makes it a promising option compared with other Zn-based aqueous batteries (≈ 0.69–1.5 V). However, due to the low utilization of Ni electrode under traditional redox reactions, widespread adoption of nickel-zinc batteries remains a challenge [13].

The nanoengineering of nickel-based cathodes has been demonstrated to be an effective approach for enhancing electrode utilization. For example, Zhou et al*.* used NiS nanodots and abundant mesopores with uniform and robust adherence that enables permeating the matrix of microspheres. The improved proton-diffusion kinetic endows Ni||Zn battery with ultrahigh areal capacity of 41.3 mAh cm^−2^ [14]. Chen et al*.* designed a unique 3D hierarchical architecture consisting of nanoscale sheets and microscale supporting skeletons, enabling the effective exposure of electrochemical active materials [11]. The prepared nickel–cobalt double hydroxides (NiCo-DHs) electrode obtained a high specific capacity of 306 mAh g^−1^. Zhou et al*.* constructed a Ni metal–organic framework as a conductive scaffold for NiMoO_4_ nanowires, achieving an exceptional capacity of 229.2 mAh g^−1^ [15]. Surface modification is also a feasible strategy to improve interfacial reactivity. For example, Yao et al*.* constructed oxygen-rich defects on Ni nanotube arrays to modulate the surface electronic structure, thereby exhibiting strong OH^−^ adsorption and achieving a high capacity of 334.9 mAh g^−1^ [16]. He et al*.* successfully manipulated the bimetallic sulfide nanointerfaces through water invoking interface corrosion, achieving a 200% increase in the capacity of electrodes [13]. In general, these strategies are based on traditional single-electron redox reactions for increasing the utilization rate of electrodes. Specifically, the improvement of electrochemical performance is achieved by compounding suitable material phases and structures, designing heterostructures as well as hierarchical nanomaterials, combining with carbon or other transition metal oxides and so on [11]. However, these nanoengineering strategies for Ni electrodes using traditional single-electron reactions can only approach their capacity limits, rather than going beyond the confines of the single-electron process.

Recently, there has been a surge of interest in the development of novel reaction mechanisms for nickel-based cathodes. For example, Xie et al*.* leveraged the oxygen produced from side oxygen evolution reaction to compensate for the coulombic efficiency (CE) loss from nickel hydroxide, thereby exhibiting an ultrahigh energy efficiency of 85% over 100 cycles at 2 mA cm^−2^ [17]. Kang et al*.* developed a two-electron transfer process including the ${\text{Ni}}^{{{2} + }} \to {\text{Ni}}^{{{3} + }}$ component and an additional ${\text{Ni}}^{{{3} + }} \to {\text{Ni}}^{{{4} + }}$ component in Ni(OH)_2_ monolayer nanosheets, delivering an exceptional redox capacity of ~ 576 mAh g^−1^, almost two times that from the one-electron process [18]. These proposed innovative reaction mechanisms have fundamentally surpassed the theoretical limits and electrode utilization of single-electron nickel batteries. However, the multi-electron techniques are still in their infancy especially for Ni electrodes, where there are considerable limitations in material composition, fabrication complexity, and achievable performance, therefore requiring further investigation.

**Scheme 1:**
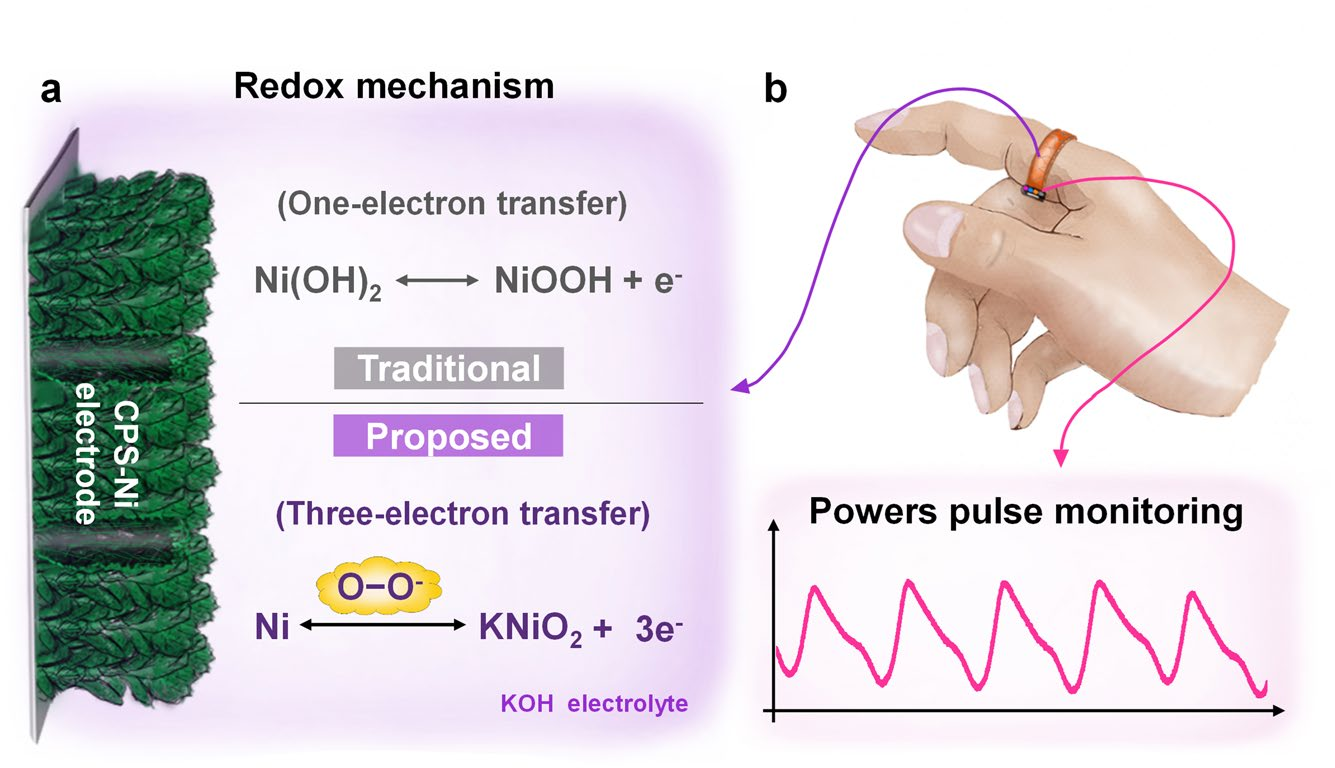
**a** mechanism of the proposed three-electron redox reaction of the CPS-Ni electrodes as compared to a traditional single-electron reaction, **b** potential application as a long-term power source in flexible electronics for vein PPG waveform monitoring

Herein, we propose a novel three-electron transfer redox mechanism based on a superoxide-activated Ni substrate that is designed to be directly and reversibly oxidized to Ni^3+^, thereby realizing Ni||Zn batteries with simultaneous ultrahigh energy and power densities (Scheme 1). To our knowledge, the achieved combined power density and energy density are unprecedented among reported Ni-based electrodes. The proposed strategy achieves several key advantages: (1) redox mechanism of three-electron transfer—high electrode utilization: the superoxide-activated Ni substrate directly participates in redox (Ni ↔ Ni^3+^), accompanied three-electron transfer, supported by *in-situ* Raman and density functional theory (DFT) simulations. (2) hierarchical nanoporous structure—efficient electrode–electrolyte interface: the high electrolyte permeability caused by capillary forces in the nanoporous structure promotes rapid ions diffusion and abundant active sites formation. As a result, the prepared CPS-Ni electrodes exhibit high energy storage performance and excellent cycling stability, namely a capacity of 3.21 mAh cm^−2^ at the current density of 5 mA cm^−2^, nearly 8 times that of the traditional cyclic voltammetry (CV) activation method. Even under the ultrahigh 200 mA cm^−2^ current density, the CPS-Ni electrodes exhibit a cycle life of more than 10,000 cycles, a high-capacity retention rate of 86.4%, and a high CE of 99.2%. The as-prepared CPS-Ni||Zn full cell is found to deliver an exceptional energy density of 6.88 mWh cm^−2^ and a power density of 339.56 mW cm^−2^. For wearable application demonstration, the CPS-Ni||Zn microbattery as an energy supply was integrated into a multi-wavelength photoplethysmography (PPG) monitoring wearable device to continuously monitor the PPG waveform for more than 7 days of a human subject. The proposed superoxide-activated electrode activation strategy opens doors to simultaneous high power and energy densities through intrinsic electron transfer improvement for Ni-based compact energy storage devices.

## Experimental Section

### Chemicals

Nickel chloride hexahydrate (NiCl_2_·6H_2_O), ammonium chloride (NH_4_Cl), sodium chloride (NaCl), potassium hydroxide (KOH), zinc oxide (ZnO) sodium polyacrylate were purchased from Shanghai Macklin Biochemical Co., Ltd.

### Fabrication of CPS-Ni Microelectrodes and the Activation Methods

CHI 760E electrochemical workstation (CH Instruments, China) was utilized for the electrochemical operation. In brief, the customized interdigital nickel plate (thickness = 0.03 mm) serves as the working electrode with saturated calomel electrode as the reference electrode, and a Pt plate as the counter electrode. The deposition process was conducted at a cathodic potential of 6 V in the mixed solution of 2 M NaCl, 2 M NH_4_Cl and 0.1 M NiCl_2_. After washing the microelectrodes with the deionized water for a few times, the microelectrodes were switched to an alkaline solution (1 M KOH) with a Hg/HgO electrode as the reference electrode. The CPS-Ni electrodes were prepared with chronopotentiometry procedure at alternative cathodic/anodic current density of 1 A cm^−2^, and the time for each process is set as 100 s. The control sample named CV-Ni electrodes was prepared with a typical cyclic voltammetry procedure within the potential range of 0.2–0.6 V at a scan rate of 10 mV s ^−1^, and the activation was finished after 1280 segments. Safety statement: chlorine and hydrogen will be released during the electrodeposition process. It is recommended to conduct the experiment in a fume hood.

### Fabrication of Zinc Microelectrode

The zinc microelectrode was obtained via the electrochemical method through three-electrode system as well. The same customized interdigital zinc plate (thickness = 0.05 mm) as working electrode is selected, the Pt plate as the counter electrode and Hg/HgO electrode as the reference electrode. The electrodeposition was conducted at a constant potential of − 1.5 V for 10 min in a solution of 6 M KOH with saturated ZnO.

### Preparation of Gel Electrolyte

Firstly, 500 mg sodium polyacrylate was slowly added into 20 mL solution of 6 M KOH with saturated ZnO. After stirring for 30 min, the transparent gel electrolyte was formed.

### Fabrication of Ni||Zn Microbatteries

Polyimide (PI) film is selected as encapsulation material. Firstly, the interdigital cathode and anode were assembled in two layers of PI film, and three edges of the two films were sealed via a sealer machine. After transferring the gel electrolyte into the encapsulated gap, the final edge was sealed and the packed Ni||Zn MB was obtained. The effective area of the interdigital Ni||Zn MB electrode is about 0.7 cm^2^, and that of the pouch battery is 4 × 4 cm^2^.

### Materials Characterization

The structure and chemical composition of the samples were characterized using FE-SEM (ZEISS Gemini 300), transmission electron microscopy with a scanning voltage of 200 kV (TEM, F200X), X-ray diffraction (XRD, Bruker discover 8 diffractometers, with Cu Kα radiation of λ = 1.5406 Å, scan range: 5°—80°), and Raman spectroscopy (WiTech alpha300R, with wavelength of 633 nm). Attenuated total refraction Fourier transform infrared spectroscopy (ATR-FTIR) (Bruker VECTOR-22, range: 400−4000 cm^−1^). The chemical states and atomic structure information were investigated by X-ray photoelectron spectroscopy (XPS) (Thermo Scientific K-Alpha), and the contact angles were measured by Dropmeter 100P.

## Results and Discussion

### Morphological Characterizations

**Fig. 1:**
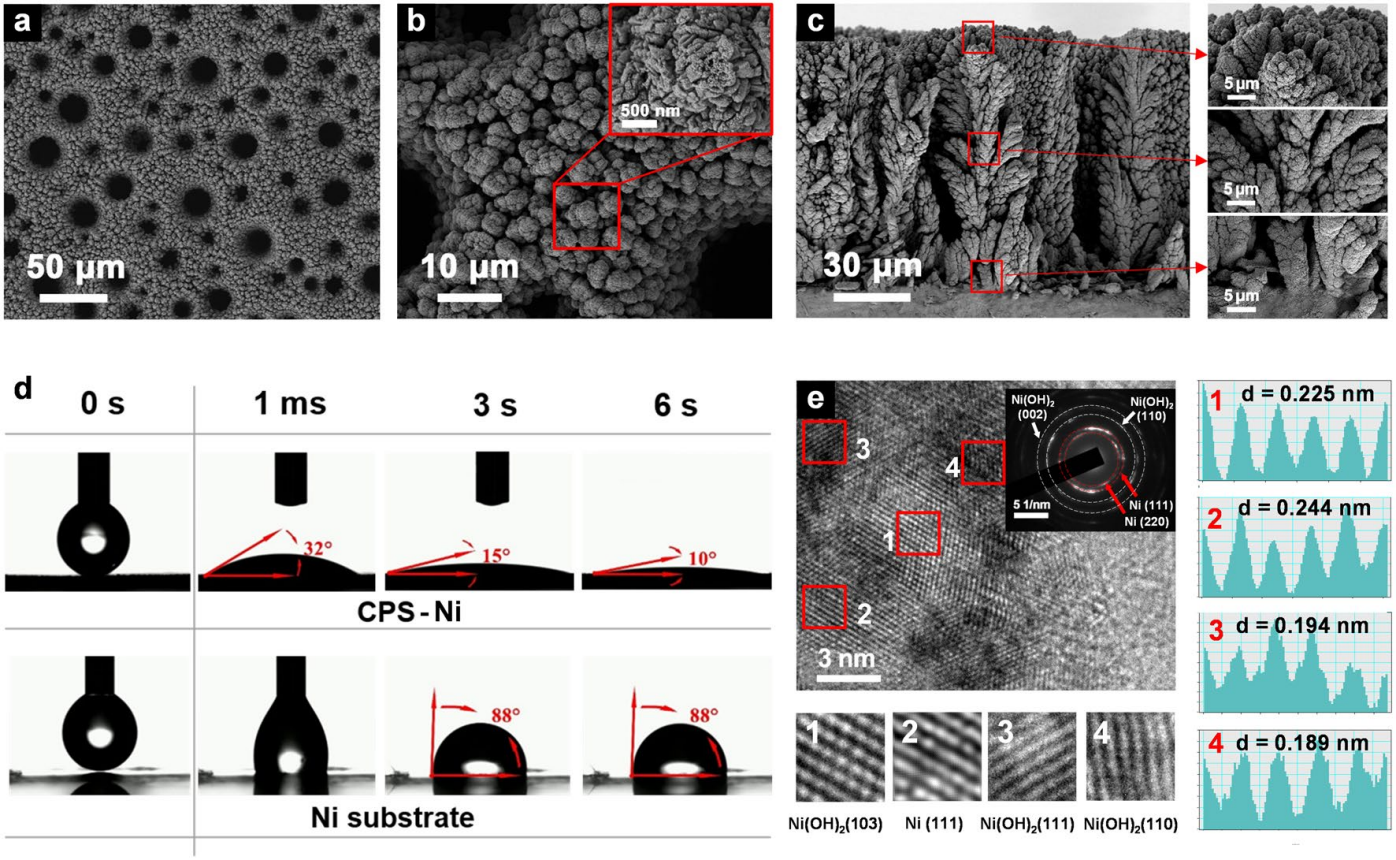
Materials characterization of the CPS-Ni electrode. SEM images showing **a**, **b** front view and **c** cross-sectional view, **d** dynamic contact angle of the CPS-Ni electrode and substrate, **e** HRTEM images and lattice plane analysis

A chronopotentiometric superoxidation method with alternating ± 1 A cm^−2^ current was employed to prepare the Ni electrodes (abbreviated as CPS-Ni electrodes, Figs. S1 and S2a). Ni electrodes prepared by the classical cyclic voltammetry method (abbreviated as CV-Ni electrodes, Fig. S2b) were used for comparison. Figure 1a, b shows the surface morphology of CPS-Ni electrodes through SEM. The CPS-Ni electrodes exhibit a regularly repeating templated microcluster structure, containing uniformly distributed pores with diameters of approximately 5 ~ 20 μm. This structure is beneficial to improving the specific surface area of the electrode and electrolyte permeability. Under the enlarged view in Fig. 1b, the electrode is composed of hierarchical nanoclusters that closely contact with each other and filled with nanometers-wide cracks. As a comparison, CV-Ni electrodes only exhibit the same macrostructure (Fig. S3), but without nanoscale cracks, which is not conducive to the penetration of electrolyte. The cross-sectional morphology of the CPS-Ni electrode (Fig. 1c) further demonstrates the composition of its microstructure. It shows lush dendrites with a height of about 100 μm growing on the Ni substrate. Partial magnification further demonstrates the strong bonding between the root of the dendrite and the substrate, which ensures structural stability during long-term cycling of the electrode. Importantly, this top-down interconnected micro-nanostructure facilitates an efficient electrolyte–electrode interface via capillary action, thereby enhancing electron/ion transport during the charging/discharging. This attribution is further supported by the dynamic contact angle of the electrode (Fig. 1d and Video S1). With only a millisecond-level contact, the CPS-Ni electrode is absorbed and penetrated by the electrolyte under capillary drive, and the contact angle quickly drops to a low 15 degrees, and stabilizes at 10 degrees after 6 s. The EDS line scan further analyzed the element distribution of the electrode. The Ni, O elements of the CPS-Ni and CV-Ni electrode (Figs. S4–S6) are evenly distributed from the electrode surface to the bottom.

Phase identification was conducted through high-resolution transmission electron microscopy (HRTEM). In Fig. 1e**,** the prepared porous hierarchical CPS-Ni electrodes exhibit the lattice fringe spacing of 0.225, 0.244, and 0.189 nm, corresponding to (103), (111), and (110) crystal plane of Ni(OH)_2_. The lattice fringe spacing of 0.244 nm corresponds to (111) crystal plane of Ni. In addition, the corresponding selected-area electron diffraction (SAED) pattern reveals a polycrystalline structure, which can be attributed to the (002), (110) planes of Ni(OH)_2_ and (111), (220) planes of Ni. The surface phase composition of CPS-Ni and CV-Ni electrodes was further confirmed using X-ray diffraction (XRD). As shown in Fig. S7a, three intense peaks at 44.5°, 51.8°, and 76.4° can be accordingly indexed to the nickel (JCPDS No. 01–070-0989), these peaks can be attributed to Ni deposition in the first preparation step. Generally, XRD measurements can only detect limited surface phases of thin-layer microelectrodes due to limited active loading. However, it is worth noting that the peaks of the new phase appeared on the CPS-Ni electrode (Fig. S7b), where the zoom-in view shows that the weak peaks at 11.6° and 33.1° can be, respectively, indexed to Ni(OH)_2_ and related hydrate (JCPDS No. 00-001-1047, No. 00–022-0444), and the peaks at 26.0° and 28.2° can be indexed to NiOOH (JCPDS No. 00-027-0956, No. 00-006-0075). The detected NiOOH and Ni(OH)_2_ species are active reactants during the charge/discharge process, which suggests that the activation depth of the CPS-Ni electrode is higher than that of the CV-Ni electrode [19].

**Fig. 2:**
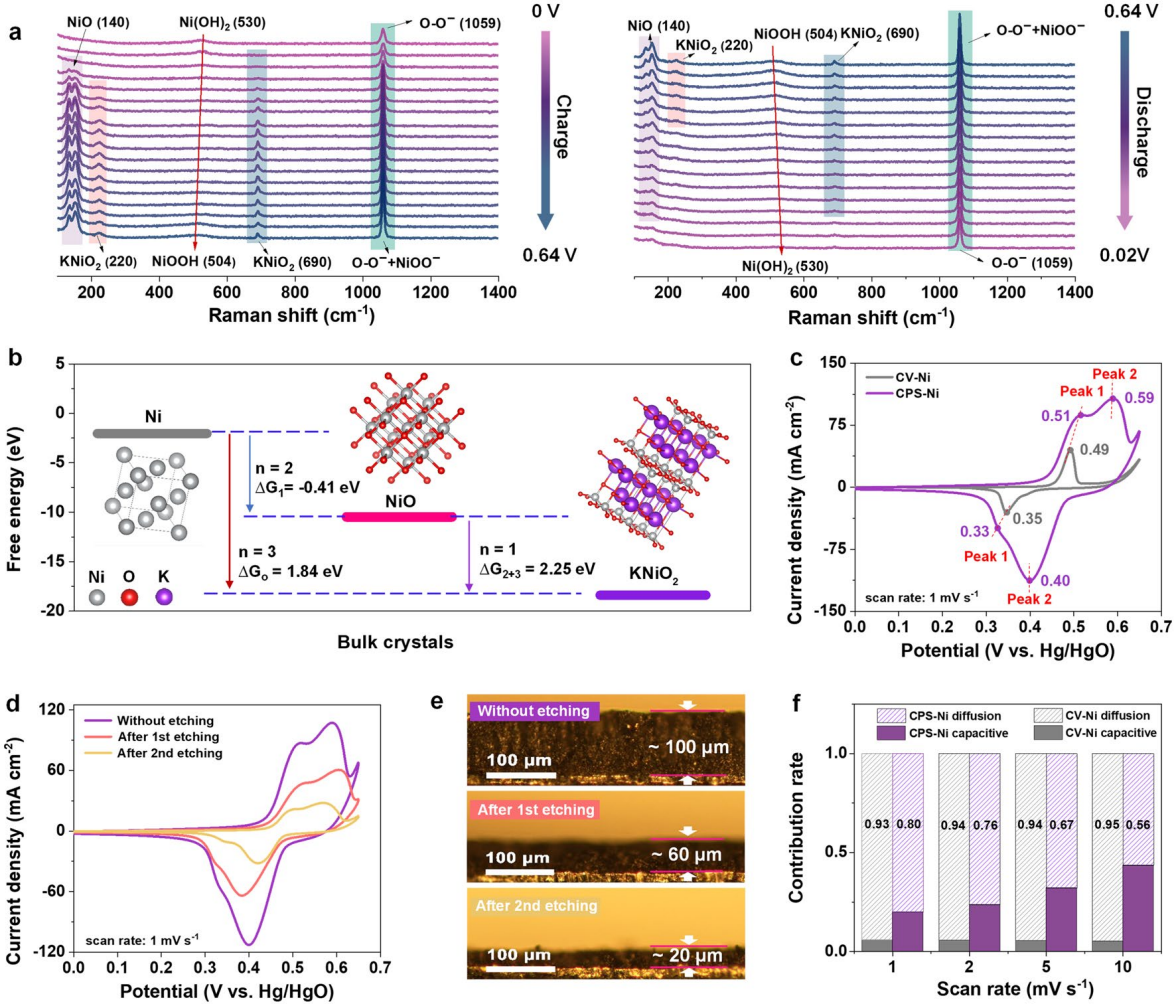
Redox mechanism of CPS-Ni electrodes. **a** In-situ Raman spectroscopy of CPS-Ni electrodes under 0–0.64 V voltage window, **b** calculated free energy of different bulk crystals and Gibbs free energy of the reaction path, **c** CV curves of CV-Ni and CPS-Ni, **d** CV curves of CPS-Ni electrodes with different etching thicknesses and **e** the corresponding optical photograph. **f** Calculated capacitive contribution of CV-Ni and CPS-Ni electrodes

XPS was used to further investigate the chemical species of two electrodes (Fig. S8). The wide-scan XPS spectra of CPS-Ni and CV-Ni electrodes all show the characteristic peaks of Ni 2*p*, O 1*s*, and C 1*s*. The oxygen content of CPS-Ni electrodes increases by 4% compared to CV-Ni electrodes, which is attributed to the contribution of the superoxide (confirmed by in-situ Raman in Fig. 2a). Furthermore, a new K elemental peak appears in CPS-Ni electrodes with 3.34% atomic proportion. In Fig. S9, high-resolution spectrum has two peaks at 292.8 and 295.6 eV corresponding to K 2*p*_3/2_ and K 2*p*_1/2_, which indicates that K compounds are generated in the electrode, which are attributed to the production of KNiO_2_ (Specific formation process in Fig. 2) [20]. In the Ni 2*p*_3/2_ high-resolution spectrum (Fig. S10), the binding energies situated at 855.6 and 856.4 eV can be assigned to the spine-orbit characteristics of Ni^2+^ and Ni^3+^ ions, respectively, while the two peaks at 861.6 and 865.4 eV are related to the shake-up satellite peak (Sat.) [21, 22]. The integrated area of the Ni^3+^ characteristic peak of CPS-Ni is about 20% higher than that of the CV-Ni electrodes, which may due to the new compound formed by K and trivalent nickel (Further discussion in Fig. 2).

### Redox Characterization and Mechanistic Description

To investigate the redox reaction mechanism of the CPS-Ni electrode, *in-situ* Raman spectroscopy has been performed under the cyclic voltammetry (CV) process. Data were collected in 20 mV steps within the voltage windows of 0 – 0.64 V (Figs. 2a and S11). At the initial 0 V, a Raman modes center at 530 cm^−1^ could be ascribed as the A_2u_(T) lattice vibration of Ni(OH)_2_ [23]. The corresponding peak weakened with further increase in potential and gradually disappeared at 0.32 V. With the potential continuedly increasing to 0.64 V, a new broad Raman band appears at 504 cm^−1^, which could be assigned to the mode of Ni–O in NiOOH [24]. This band shift is consistent with the reported conventional electrochemical oxidation of Ni(OH)_2_–NiOOH [11], as described in Eq. (1):

1 $${\text{Ni}}\left( {{\text{OH}}} \right)_{{2}} + {\text{ OH}}^{ - }= \, {\text{NiOOH }} + {\text{ e}}^{ - } + {\text{ H}}_{{2}} {\text{O}}$$

A characteristic Raman band appears at 1059 cm^−1^ under the initial 0 V, which could be assigned to superoxide groups (O–O^−^) that resulted from the highly oxidative applied current during the activation process [25], which was also verified by FTIR results (Fig. S12). As the voltage increases to 0.12 V, a new Raman band at 140 cm^−1^ appears, which could be assigned to NiO [26]. Simultaneously, a pairs of Raman bands appear at 220 and 690 cm^−1^ could be identified as KNiO_2_ [22, 27, 28], which was also verified by XRD pattern (Fig. S13). When the voltage rises from 0.12 to 0.64 V, the intensity of NiO, KNiO_2_ obviously increases without Raman shift. Furthermore, the Raman bands at 1059 cm^−1^ also gradually increased, which was explained in previous studies as the negatively charged pernickelate (NiOO^−^) [11]. Compared with conventional Eq. (1) consisting only Ni(OH)_2_ and NiOOH phases, four newly produced species, namely O–O^−^, NiOO^−^, NiO, and KNiO_2_, suggesting a new redox mechanism during the charge process of CPS-Ni electrodes. Furthermore, we used the unactivated Ni substrate as the electrode and tested the CV and charge–discharge curve in KOH electrolyte containing superoxide radicals. The results verified the fact that KNiO_2_ was generated on the Ni substrate only under the activation of superoxide radicals. Details are shown in Figs. S14–S17. Combined with the results of Raman spectroscopy, we propose a redox reaction containing three-electron transfer in CPS-Ni electrode system, as described in Eq. (2):

2 $$\text{Ni } \stackrel{\text{ O}{\text{O}}^{-},\text{ KOH }}{\longleftrightarrow} \text{ KNiO}_2 + 3\text{e}^-$$

This reaction reveals that the three-electron transfer of the Ni redox reaction participated by K under the catalysis of O–O^−^, which is further confirmed by XPS spectra of the K (2*p*) and Ni (2*p*) regions (details in Figs. S9 and S10). During the discharge process (Fig. 2a, right), the signals of NiOOH gradually disappear, replaced by a broad Raman band at 530 cm^−1^, suggesting reaction reversibility of Ni(OH)_2_ ↔ NiOOH (Eq. (1)). Simultaneously, the intensity of the KNiO_2_ (220 and 690 cm^−1^) has gradually decreased and eventually reduced to nickel metal, which indicates the reversibility of Ni ↔ KNiO_2_ (Eq. (2)). Furthermore, during the whole CV cycle, the superoxide signals (1059 cm^−1^) exist unanimously, which suggests that it has not been exhausted under the redox reaction. Previous studies reported that KNiO_2_ could be formed through the reaction of K_2_O_2_ and NiO, which led to the stabilization of nickel in the trivalent formal oxidation state [27, 29]. However, those reactants were not evident in the Raman results, perhaps due to the transient nature of its instability. Based on the products and reactants that have been characterized, we proposed the following most likely specific reaction of Eq. (2):

$$2\text{Ni }+ 2\text{KOH }+ 2\text{H}_2\text{O } \begin{array}{c}\stackrel{{\text{ O}{\text{O}}^{-}}_{\left(cat\right) } }{\longleftrightarrow }\\ \end{array}\;2\text{KNiO}_2 + 3\text{H}_2 \;(\text{overall reaction})$$

$${\text{Ni}}\, + \,{\text{H}}_{{2}} {\text{O}}\, = \,{\text{2H}}^{ + } \, + \,{\text{2e}}^{ - } \, + \,{\text{NiO }}({\text{Step 1}})$$

$${\text{H}}_{{2}} {\text{O}}\, + \,{\text{NiO}}\, = \,{\text{H}}^{ + } \, + \,{\text{e}}^{ - } \, + \,{\text{NiHOO }}({\text{Step 2}})$$

$${\text{NiHOO}}\, + \,{\text{KOH}}\, = \,{\text{KNiO}}_{{2}} \, + \,{\text{H}}_{{2}} {\text{O }}({\text{Step 3}})$$

DFT was used to optimize the crystal structures of reactants and products (Fig. 2b and Table S1), and the Gibbs free energy of the overall reaction (ΔG_o_) and sub-reactions (ΔG_1,_ ΔG_2,_ ΔG_3_) without electric field was calculated (Table S1). The results show that the ΔG_o_ is 1.84 eV, the ΔG_1_ is − 0.41 eV, the ΔG_2_ is 2.84 eV, and the ΔG_3_ is − 0.59 eV. This illustrates that the overall reaction and step 2 are not likely to occur without the action of an electric field. The Gibbs free energy of each step at 0.59 V versus Hg/HgO (peak voltage from CV curve) was calculated using the formula G(U) = G(0 V) – neU, where e is the elementary charge of an electron, n is the number of proton-electron pairs transferred, and U is the applied potential (vs RHE). The calculated results show that ΔG_o_(0.59 V) = -5.44 eV, ΔG_1_(0.59 V) = − 5.26 eV, ΔG_2_(0.59 V) = 0.415 eV, ΔG_3_(0.59 V) = − 0.593 eV. The above calculation results show that the overall reaction is possible at a voltage of 0.59 V, while the step 2 is difficult to occur even under this electric field. Therefore, combined with the actual reaction results, it can be inferred that superoxide may act as a catalyst to reduce the ΔG_2_, resulting in the step 2 reaction being able to proceed. This is also consistent with the result that pernickelate (NiOO^−^) form step 2 was detected by Raman spectroscopy.

The CV curves of CV-Ni electrode and CPS-Ni electrode at the scan rate of 1 mV S^−1^ are shown in Fig. 2c. The CV-Ni electrodes show oxidation and reduction peaks (peak 1) at 0.49 and 0.35 V, respectively, which are attributed to the classic single-electron reaction between Ni(OH)_2_ and NiOOH [30]. As a comparison, the CPS-Ni electrodes show two obvious pairs of redox peaks appear at 0.51/0.33 V (peak 1) and 0.59/0.40 V (peak 2). Specifically, the peak 1 of redox potentials at 0.51/0.33 V consists of the classical reaction mentioned in the CV-Ni electrode. The peak 2 of redox potentials at higher potentials of 0.59/0.40 V could be assigned to the new redox reaction of Ni and KNiO_2_, which is consistent with the *in-situ* Raman results during the CV charge/discharge process. Furthermore, the higher peak current density of peak 1 (86 mA cm^−2^) and peak 2 (107 mA cm^−2^) of the CPS-Ni electrodes compared with CV-Ni (45 mA cm^−2^, peak 1) reveals its more electrochemical capacity contribution. The corresponding integrated area of the CV curves, the capacity of the CPS-Ni electrodes (5.0 mAh cm^−2^) is almost 8 times that of the CV-Ni electrodes (0.63 mAh cm^−2^). This means that the oxidation and the reduction peak of the CPS-Ni electrodes should be the superposition of multiple electron transfer, which is consistent with our proposed Eq. (2) The selected-area Raman mapping of CPS-Ni electrodes (Fig. S18) shows the characteristic peak and distribution of Ni(OH)_2_ and superoxide species [24, 31]. The brighter superoxide signal exhibits a denser distribution than that of Ni(OH)_2_, which is consistent with the peak intensity of the two groups of redox reaction in CV curves (Fig. 2c). Compared with CPS-Ni electrodes, the Raman result of CV-Ni electrodes (Fig. S19) only shows one characteristic peak of Ni(OH)_2_ at 579 cm^−1^.

To study the electrode reaction depth of superoxide participation and the capacity contribution by hierarchical nanoporous structure, three different thicknesses (100, 60, and 20 μm) of CPS-Ni electrodes were prepared by laser etching (Fig. 2d, e). XRD and XPS data confirmed the phase integrity of the electrode materials (Figs. S20 and S21). The CV curves of the three electrodes with different thicknesses at 1 mV s^−1^ all show two redox peaks, and the peak current density decreases with decreasing thickness (Fig. 2d). These results indicate that superoxide is deeply involved in the reaction of the entire electrode, not just a surface reaction behavior. Even after two times of etching (thicknesses ≈ 20 μm), the integral capacity (1.2 mAh cm^−2^) of CV curves still indicates a performance around double that of the CV-Ni electrode (0.63 mAh cm^−2^, thicknesses ≈ 100 μm), which further confirms the high electrode utilization of superoxide-activated Ni substrate directly involved in redox reaction. To analyze the ion diffusion behavior in the Ni electrodes, the cyclic voltammetry curves have been performed at scan rates ranging from 0.5 to 10 mV s^−1^ (Fig. S22). Although the diffusions of both Ni electrodes were mixed processes by capacitive and diffusion processes, the CPS-Ni electrodes exhibit stronger capacitance behavior. Quantificationally, according to $i={k}_{1}v+{k}_{2}{v}^{0.5}$, where *k*_*1*_*v* and *k*_*2*_*v*^*0.5*^ represent the pseudo-capacitive and ionic diffusion contributions, respectively [32]. As the scan rate increases from 1 to 10 mV s^−1^, as shown in Fig. 2f, the pseudo-capacitance contribution of the CPS-Ni electrodes gradually increases from 20% to 44%, which is higher than that of the CV-Ni electrodes, mainly attributed to the rapid ion diffusion (capillary behavior) and high specific surface area caused by the hierarchical nanoporous structure of the CPS-Ni electrodes. The electrochemical active surface area (ECSA) of the two electrodes was analyzed by the CV method. As shown in Fig. S23, the proposed CPS-Ni electrode exhibits a double-layer capacitance of 78.04 mF cm^−2^, which was significantly higher than that of the CV activation methods (CV-Ni 12.63 mF cm^−2^). Combined with SEM images and dynamic contact angles, the rich electrochemical active sites brought by the high specific surface area of the CPS-Ni electrode were further verified.

Based on the above results, the electrochemical behaviors and reaction mechanisms of the CPS-Ni electrode are summarized as follows: (1) redox mechanism of three-electron transfer leads to high electrode utilization. The superoxide-activated Ni substrate directly participates in redox (Ni ↔ Ni^3+^), achieving three-electron transfer. (2) hierarchical nanoporous structure leads to an efficient electrode–electrolyte interface. The high electrolyte permeability caused by capillary forces in the nanoporous structure facilitates rapid ions diffusion and abundant active site formation.

### Electrochemical Performance

**Fig. 3:**
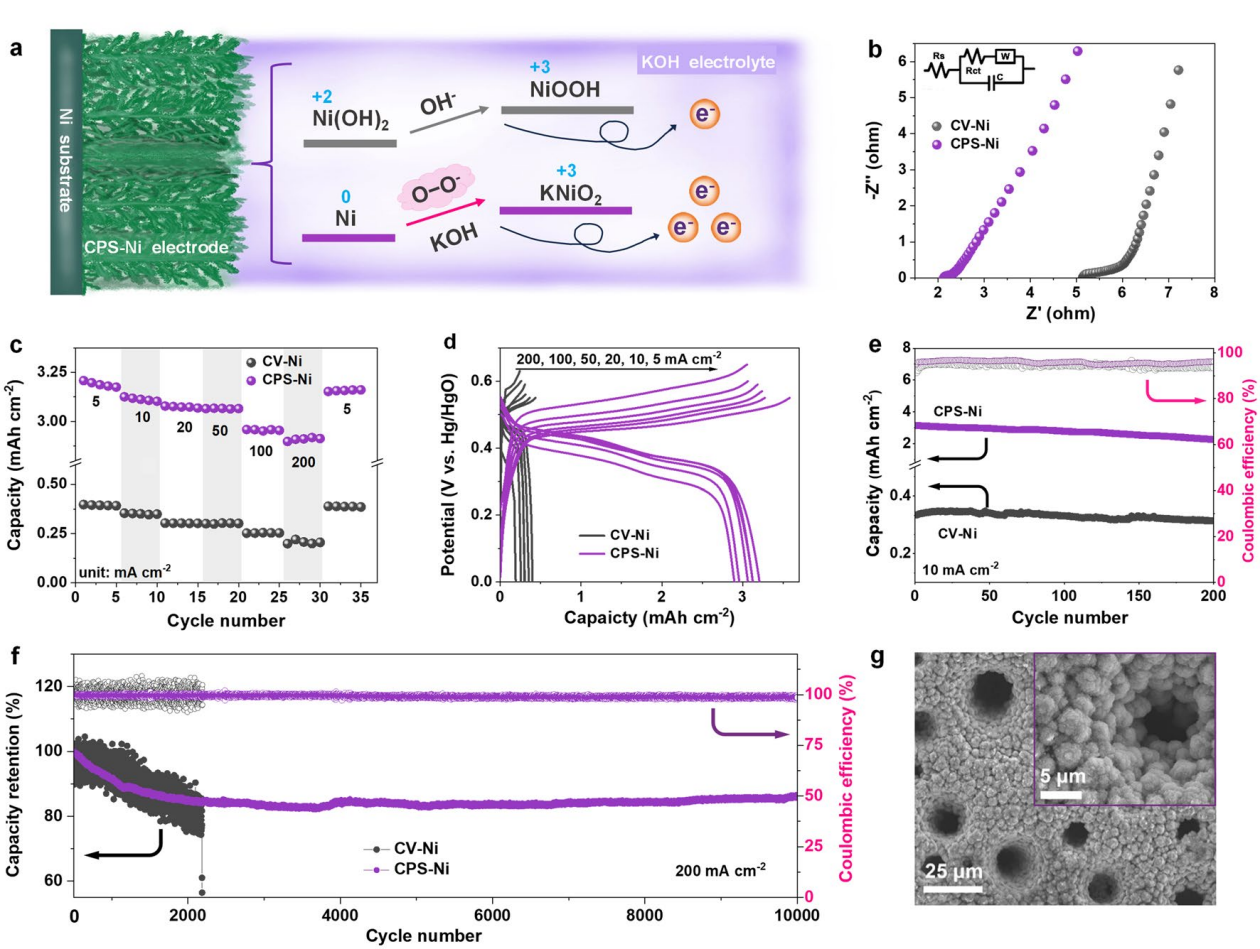
Electrochemical performance of different electrodes under asymmetric half-cells. **a** Schematic of redox reaction of CPS-Ni electrode in KOH electrolyte, **b** EIS curves, **c** rate capacity, **d** charge and discharge curves, **e** cycling performance at 10 mA cm^−2^, **f** long-term cycling performance at 200 mA cm^−2^ of CPS-Ni electrode and CV-Ni electrode, **g** SEM images of CPS-Ni electrode after 10,000 cycles

Fig. 3a visualizes the mixed two redox reaction processes of CPS-Ni electrodes with a hierarchical nanoporous structure in KOH electrolyte. First, there is the classic redox reaction between Ni(OH)_2_ and NiOOH, with a single-electron transfer. Second, under the activation of superoxide radical activation, the redox reaction between Ni metal and KNiO_2_ occurs in the KOH electrolyte, facilitating three-electron transfer. Profiting from the capillary action of the CPS-Ni electrode [33], CPS-Ni exhibits a lower charge transfer resistance of 2.1 Ω (Figs. 3b and S24), compared to CV-Ni (5.2 Ω). The rate performance of CPS-Ni electrode was evaluated at the current density from 5 to 200 mA cm^−2^ within a three-electrode system. As shown in Fig. 3c, CPS-Ni displays an excellent capacity of 3.21 mAh cm^−2^ at the current density of 5 mA cm^−2^, which is more than 8 times that of CV-Ni electrodes (0.40 mAh cm^−2^). When the current density increases to 10, 20, 50, 100, and 200 mA cm^−2^, the capacities of CPS-Ni are 3.13, 3.08, 3.07, 2.96, and 2.92 mAh cm^−2^, respectively, with a capacity retention of 91.0%. As a comparison, under the same current density, the capacities of CV-Ni electrodes are 0.35, 0.30, 0.29, 0.25, and 0.21 mAh cm^−2^, respectively. The capacity of CV-Ni electrodes retention rate is only 52.5%, which may be due to the shallow and unstable-activated layer that cannot withstand a high current, especially at the level of 200 mA cm^−2^ [30]. When the current density returned to 5 mA cm^−2^, CPS-Ni electrodes still show a high capacity of 3.16 mAh cm^−2^, compared to CV-Ni electrodes (0.39 mAh cm^−2^). The ultrahigh discharge capacity and excellent rate performance of CPS-Ni electrodes are attributed to the improved electrode utilization due to the hierarchical nanoporous structure and the three-electron transfer between Ni and KNiO_2_. The galvanostatic charge/discharge (GCD) curves of CPS-Ni and CV-Ni electrodes at different specific current within 0–0.65 V are shown in Fig. 3d. Compared to the CV-Ni electrodes of the classical single-step discharge plateau (0.45 V), CPS-Ni electrodes all show two-step and longer discharge time (0.45 and 0.36 V), representing two redox capacities, which is consistent with the CV curves.

The long-term cycling performance of the two electrodes at different current densities further evaluates electrode utilization and stability. As shown in Fig. 3e, after 200 cycles at current density of 10 mA cm^−2^, the specific capacity of CPS-Ni electrodes maintains from 3.13 mAh cm^−2^ (1st cycle) to 2.25 mAh cm^−2^ (200th cycle) with the average coulombic efficiency (CE) of 95.9%, while CV-Ni electrodes maintain from 0.33 to 0.31 mAh cm^−2^ with a lower average CE of 94.4%. Noteworthily, even under the ultrahigh 200 mA cm^−2^ current density (Fig. 3f), the CPS-Ni electrodes exhibit a cycle life of more than 10,000 cycles, a high-capacity retention rate of 86.4%, and a high average CE of 99.2%. In contrast, the capacity of the CV-Ni electrode drops sharply after only 2180 cycles, accompanied by extremely fluctuating CE. This is because the CV-Ni electrode only has the classic Ni(OH)_2_ $\leftrightarrow$ NiOOH electrochemical process, and the active material Ni(OH)_2_ and NiOOH are easily broken down under high current conditions, causing irreversible structural collapse with repeated lattice strain [30]. In the CPS-Ni electrode, not only does the Ni(OH)_2_ $\leftrightarrow$ NiOOH reaction occur, but Ni also directly participates in the electrochemical redox process under the stimulation of the superoxide, ensuring full utilization of the Ni substrate, thereby achieving high capacity of the electrode and maintaining structural stability under long cycles. As evident in Fig. 3g, the SEM images of the CPS-Ni electrode after 10,000 cycles show that the porous hierarchical structures of the electrode are completely retained, and the nanostructures are tightly connected without obvious defects resulting. In contrast, the CV-Ni electrode exhibits a loose and damaged structure (Fig. S25). These above results suggest that the novel three-electron redox mechanism of Ni $\leftrightarrow$ KNiO_2_ activated by the superoxide can effectively improve electrode utilization, capacity, and long-cycle stability.

### Practical Application

**Fig. 4:**
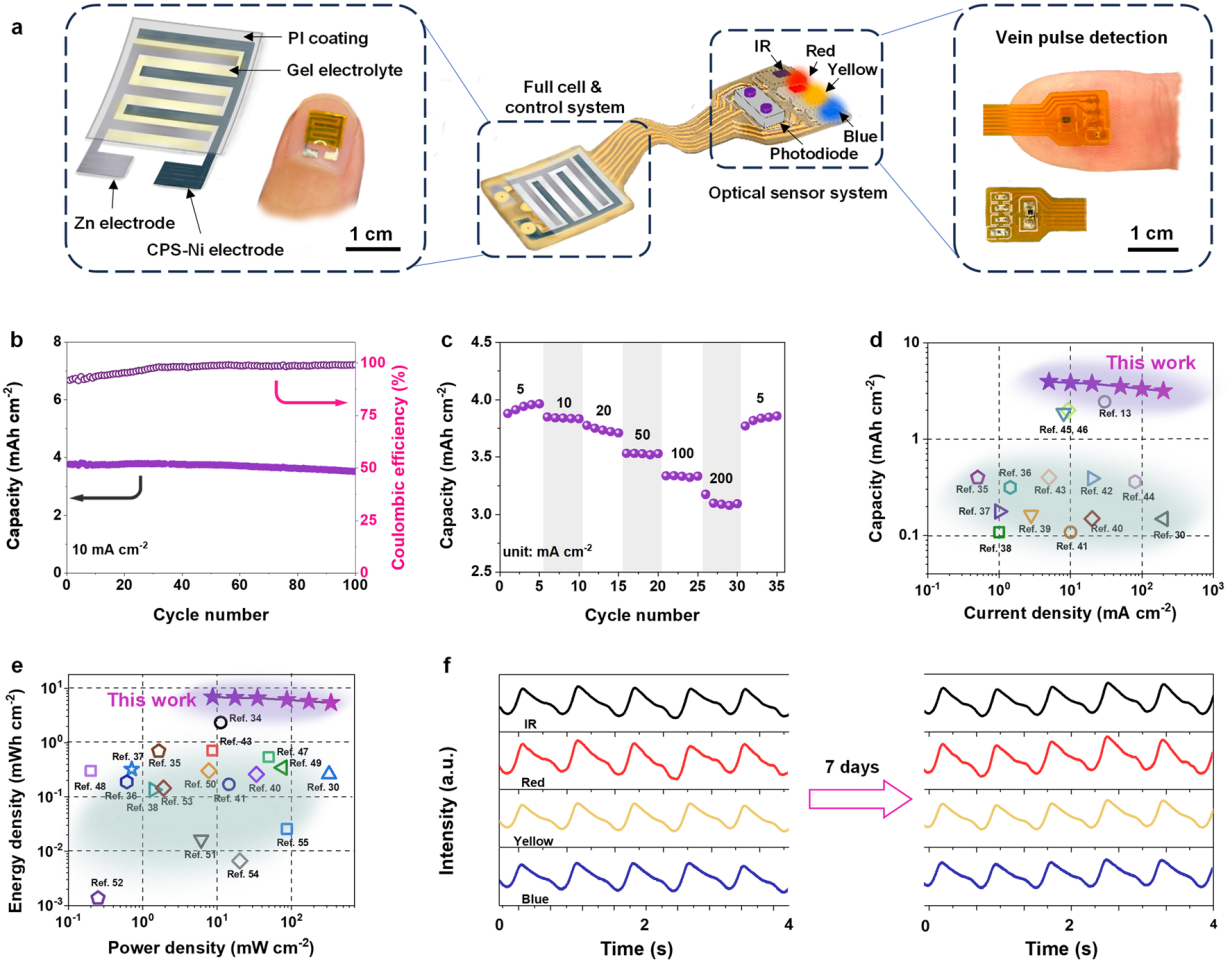
Electrochemical performance and wearable applications of CPS-Ni||Zn full cell. **a** Schematic of CPS-Ni||Zn microbatteries integrated into a wearable vein PPG waveform detection device, **b** cycling performance at 10 mA cm^−2^, **c** rate capacity, **d** capacity and **e** energy/power density comparison against recently reported high-performance micro-power sources, **f** blood pulse signals detected using the various on-board light sources

As a demonstration of the proposed strategy in a practical application, a Ni||Zn microbattery was assembled using Zn as the anode and CPS-Ni electrode as the cathode (CPS-Ni||Zn MB). As shown in Fig. 4a, the microcell with interdigitated electrodes has an area of only 0.7 cm^2^ and a thickness of only 0.5 mm. This small size and bendable characteristics enable the microbattery to be highly flexible. As shown in Figs. S26 and S27, the equivalent resistance of CPS-Ni||Zn MB is only 1.53 Ω, and the CV curves exhibit repetitive and stable redox peaks with the 1.3–2.1 V voltage window under 0.5–10 mV s^−1^ rate scan. Figure 4b shows the cycling performance of CPS-Ni||Zn MB at the current density of 10 mA cm^−2^. The initial capacity is 3.76 mAh cm^−2^, and the capacity after 100 cycles is 3.52 mAh cm^−2^, with a high-capacity retention rate of 93.6%. The pouch-packed CPS-Ni||Zn full battery also verified the capacity and stability of the CPS-Ni electrodes (Fig. S28). As shown in Fig. 4c of the rate performance, the capacity of the CPS-Ni||Zn MB can reach 3.96, 3.85, 3.78, 3.53, 3.34, and 3.18 mAh cm^−2^ at the current densities of 5, 10, 20, 50, 100, and 200 mA cm^−2^, respectively, exhibiting an excellent rate performance of 80.3% even under a 40-time current density increase. Furthermore, when the current density returns to 5 mA cm^−2^ after 30 cycles, the specific discharge capacity returns to 3.86 mAh cm^−2^. GCD curves of CPS-Ni||Zn MBs are presented in Fig. S29, showing a high operating voltage plateau of about 1.72–1.78 V, which arises from high reversibility of metallic nickel during the secondary electrochemical process and the high reactive activity provided by hierarchical porous structure. The above results indicate the high electrode utilization, excellent cycling stability and electrochemical reversibility of CPS-Ni||Zn MB. The performance metrics of the CPS-Ni cathode and CPS-Ni||Zn full cell, including the specific capacity, rate capability, power density, and energy density, were compared against other Ni base cathodes (Fig. 4d, e, Table S2) [13, 30, 34–54]. In particular, the capacity of our work (3.96 mAh cm^−2^ at current density of 5 mA cm^−2^) is about 10 times that of the best work (0.4 mAh cm^−2^) that has been reported for aqueous Ni–Zn batteries with one-electron transfer, which is remarkable [13]. The CPS-Ni||Zn MB optimal energy density of 6.88 mWh cm^−2^ and power density of 339.56 mW cm^−2^, which exhibit competitive performance to the point it rivals that of commercial lithium batteries [35, 48, 49], as shown in Table S3.

To verify the commercial feasibility of a CPS-Ni||Zn MB as a wearable energy storage device, the CPS-Ni||Zn MB as a power source was integrated into multi-wavelength photoplethysmography wearable electronics (MWPPG) to measure the continuous PPG waveform. As shown in Figs. 4a and S30, the MWPPG integrates light emitting diode (infrared, red, yellow, blue LED) photodetector, CPS-Ni||Zn MB and control system. The sensors can be mounted on a fingertip to record data in real time at a sampling rate of 1 kHz. The four LEDs were switched on for 0.25 ms in turn with a 0.05 ms interval. As shown in Fig. 4f, the human PPG waveform is continuously monitored for 7 days from a device mounted as a finger ring, which further confirms that CPS-Ni||Zn MB can operate as a long-term power source even for sensing and computation intensive wearable applications.

## Conclusion

This work describes the design, realization, and application of a superoxidation strategy for activating the Ni electrode and demonstrating a novel mechanism that facilitates the three-electron transfer redox reaction (Ni → Ni^3+^). The as-prepared CPS-Ni electrodes exhibit an ultrahigh capacity, nearly 8 times that of traditional one-electron processes electrodes. The CPS-Ni||Zn full cell delivers unprecedentedly high combined energy and power densities. The superoxidation-activated strategy functioning in a long lifetime battery is further demonstrated in wearable electronics, demonstrating its capability to power computational intensive AIoT systems such as real-time PPG waveform sensing and signal processing applications.

## Supplementary Information

Below is the link to the electronic supplementary material.

Supplementary file1 (DOCX 9259 kb)

Supplementary file2 (MP4 734 kb)
