# Supplementary material for: Ultrahigh Energy and Power Density in Ni–Zn Aqueous Battery via Superoxide-Activated Three-Electron Transfer
Source: Nanomicro Lett. 2024 Nov 29;17:79. doi: 10.1007/s40820-024-01586-z (PMC11607291; doi:10.1007/s40820-024-01586-z)
Supplement: Supplementary file 1 — Supplementary file1 (DOCX 9259 kb) [file 40820_2024_1586_MOESM1_ESM.docx]

Supporting Information for

**Ultrahigh Energy and Power Density in Ni–Zn Aqueous Battery via Superoxide‑Activated Three‑Electron Transfer**

Yixue Duan^1^, Bolong Li^2, 3^, Kai Yang^2, 3,^ *, Zheng Gong^3^, Xuqiao Peng^4^, Liang He^1,^ *, Derek Ho^2, 3,^ *

^1^ School of Mechanical Engineering, State Key Laboratory of Intelligent Construction and Healthy Operation and Maintenance of Deep Underground Engineering, Sichuan University, Chengdu 610065, P. R. China

^2^ Department of Materials Science and Engineering, City University of Hong Kong, Kowloon, Hong Kong, 999077, P. R. China

^3^ Hong Kong Centre for Cerebro-cardiovascular Health Engineering, Hong Kong Science Park, Hong Kong, 999077, P. R. China

^4^ School of Mechanical Engineering, Sichuan University, Chengdu 610065, P. R. China

*Corresponding authors. E-mail: [kyang@hkcoche.org](mailto:kyang@hkcoche.org) (Kai Yang) ; [hel20@scu.edu.cn](mailto:hel20@scu.edu.cn) (Liang He); [derekho@cityu.edu.hk](mailto:derekho@cityu.edu.hk) (Derek Ho)

**S1 Supplementary Experimental Section**

**S1.1 Electrochemical measurement**

Cyclic voltammetry (CV), Galvanostatic charge-discharge (GCD), and electrochemical impedance spectroscopy (EIS) tests were all conducted on a CHI760E. Cycling tests were performed on the LAND CT2001A battery test system (Wuhan, China). The electrochemical performance of Ni electrode was tested in a three-electrode system with CPS-Ni/CV-Ni, Pt electrode and Hg/HgO electrode as the working, counter, and reference electrodes, respectively. All the electrochemical tests related to the half-cell were conducted in 1 M KOH solution while the Ni||Zn MB was tested in the gel electrolyte of 6 M KOH with saturated ZnO. All measurements were carried out at room temperature (25 °C). The specific capacity (*C*) of the single microelectrode or assembled MB, the energy density (*E*), and the power density (*P*) of the assembled MB were calculated by the formulas *C* = *It*/*A*, *E* = $I\int_{0}^{t} V(t)dt$/*A*, and *P* = *E*/*t*, where *I* is the discharge current (mA cm^−2^), *A* is the effective area of the microelectrode or the packed MB (cm^2^), and *V(t)* is the discharge voltage (Note: there may be slight increase of the effective area after the microelectrodes were packed, and we still define it as 0.7 cm^2^). EIS measurement was conducted at an AC voltage of 5 mV amplitude with the frequency range of 100 kHz to 0.1 Hz at open circuit potential.

**S1.2 DFT Calculation**

All spin-polarized first-principle computations were performed under density functional theory (DFT). The elemental core and valence electrons were represented by the projector augmented wave (PAW) method and plane-wave basis functions with a cutoff energy of 500 eV. Generalized gradient approximation with the Perdew-Burke-Ernzerhof (GGA-PBE) exchange-correlation function was employed in all the calculations. Geometry optimizations were performed with a force convergency smaller than 0.02 eV/Å and the energy convergence of 1 × 10^-6^ eV. The DFT-D3 empirical correction method was employed to describe van der Waals interactions. The DFT + U approach was introduced to treat the highly localized Ni 3d states with parameters of U – J = 6.2 eV.

The free energy of each crystal (*G*) and Gibbs free energy of reaction (*ΔG*) are given by:

*G = E + ZPE - TS*

*ΔG = G_products_ - G_reactants_*

where *G_reactants_*, and *G_products_* denote the sum of Gibbs free energies of the product and reactant, respectively.

**S2 Supplementary Tables**

**Table S1** The calculated free energy for each reaction unit

|  | E (eV) | ZPE (eV) | TS (eV) | G (eV) | Number of units | G of a unit (eV) |
| --- | --- | --- | --- | --- | --- | --- |
| Ni | -8.923 | 0.140 | 0.210 | -8.993 | 4.000 | -2.248 |
| KNiO_2_ | -36.591 | 0.340 | 0.540 | -36.791 | 2.000 | -18.395 |
| KOH | -54.909 | 1.490 | 0.750 | -54.169 | 4.000 | -13.542 |
| NiO | -41.643 | 0.410 | 0.410 | -41.643 | 4.000 | -10.411 |
| H_2_O | -119.859 | 5.520 | 0.640 | -114.979 | 8.000 | -14.372 |
| H_2_ | -13.646 | 0.580 | 0.180 | -13.246 | 2.000 | -6.623 |

**Table S2** Comparison of highest current density and highest capacity of CPS-Ni||Zn with other batteries including aqueous system and organic system

| Electrode material | Current density  (mA cm^−2^) | Capacity  (mAh cm^−2^) | References |
| --- | --- | --- | --- |
| **Zn-based aqueous batteries** | | | |
| **CPS-Ni\|\|Zn** | **5, 10, 20, 50, 100, 200** | **3.96,** **3.85, 3.78, 3.53, 3.34, 3.18** | **This work** |
| CNSOH-1\|\|Zn@CC | 30 | 2.45 | [13] |
| Ni-Ni(OH)_2_/Zn(OH)_2_\|\|Zn | 200 | 0.149 | [30] |
| HCNT-O\|\|Zn | 10 | 1.932 | [34] |
| VO_2_(B)-MWCNTs\|\|Zn | 1.43 | 0.314 | [36] |
| α-MnS\|\|Zn | 1 | 0.178 | [37] |
| Ni-Co LDH@CC\|\|Zn | 1 | 0.109 | [38] |
| V_2_O_5_@CNTs\|\|Zn@CNTs | 2.8 | 0.164 | [39] |
| HOP Ni@Ni(OH)_2_\|\|Zn | 20 | 0.150 | [40] |
| Co(OH)_2_@NiCo LDH\|\|Zn | 10 | 0.108 | [41] |
| CC-CF@NiO\|\|CC-CF@ZnO | 20 | 0.39 | [42] |
| Ni-MOF/CNTF\|\|Zn | 5 | 0.4 | [43] |
| DHTP-Ni-MOF NFAs/CNTF\|\|Zn | 80 | 0.36 | [44] |
| MnO_2_\|\|Zn | 0.08 | 0.102 | [51] |
| **Organic batteries** | | | |
| LTO@GC\|\|LFP@GC | 0.50 | 0.398 | [35] |
| NiO@NiO\|\|Li | 9.2 | 2.0 | [45] |
| NiO@N-CNTs\|\|Li | 8.0 | 1.87 | [46] |
| MoS_2_@C\|\|Li | 5.0 | 3.428 | [S1] |
| B-SnS_2_\|\|Na | 40 | 3.7 | [12] |
| FeS@C\|\|Na | 5 | 1.24 | [13] |

**Table S3** Comparison of peak energy density and peak power density of CPS-Ni||Zn with other energy storage devices including batteries and supercapacitors

| Electrode material | Peak energy density  (mWh cm^−2^) | Peak power density  (mW cm^−2^) | References |
| --- | --- | --- | --- |
| **Zn-based aqueous batteries** | | | |
| **CPS-Ni\|\|Zn** | **6.882** | **339.56** | **This work** |
| CNSOH-1\|\|Zn | 4.29 | 8.75 | [13] |
| Ni-Ni(OH)_2_/Zn(OH)_2_\|\|Zn | 0.26 | 320.17 | [30] |
| HCNT-O\|\|Zn | 2.339 | 11.18 | [34] |
| VO_2_(B)-MWCNTs\|\|Zn | 0.189 | 0.61 | [36] |
| α-MnS\|\|Zn | 0.322 | 0.710 | [37] |
| Ni-Co LDH@CC\|\|Zn | 0.136 | 1.4 | [38] |
| HOP Ni@Ni(OH)_2_\|\|Zn | 0.26 | 33.8 | [40] |
| Co(OH)_2_@NiCo LDH\|\|Zn | 0.17 | 14.4 | [41] |
| Ni-MOF/CNTF\|\|Zn | 0.71 | 8.6 | [43] |
| NiSA-SSA 160\|\|Zn@CC | 0.54 | 49.49 | [47] |
| Ag\|\|Zn | 0.301 | 7.728 | [50] |
| MnO_2_\|\|Zn | 0.0163 | 6.1 | [51] |
| Ni-NiO\|\|Zn | 0.006 | 20.2 | [54] |
| NF@NiO\|\|Zn | 0.026 | 86.48 | [S2] |
| **Organic batteries** | | | |
| LTO@GC\|\|LFP@GC | 0.695 | 1.64 | [35] |
| BAC\|\|CoMoO_4_/RGO Li MSC | 0.301 | 0.200 | [48] |
| V_2_O_5_\|\|Li | 0.345 | 75.5 | [49] |
| NVP\|\|NTO NIMB | 0.145 | 1.9 | [53] |
| NVPF\|\|NaBF_4_-IE//NTP | 7.33 | 7.11 | [S3] |
| LiFePO_4_\|\|Li | 29.1 | 12.7 | [S4] |

**S3 Supplementary Figures**

**Fig. S1 a** The scheme of CPS-Ni electrodes preparation and **b** the corresponding chronopotentiometry curves

**Fig. S2** Electrochemical activation curves of **a** CPS-Ni and **b** CV-Ni electrodes

**Fig. S3 a** Surface SEM images of CV-Ni electrodes and **b** enlarged views

**Fig. S4** The cross-sectional view with the EDS line scan of CPS-Ni electrodes


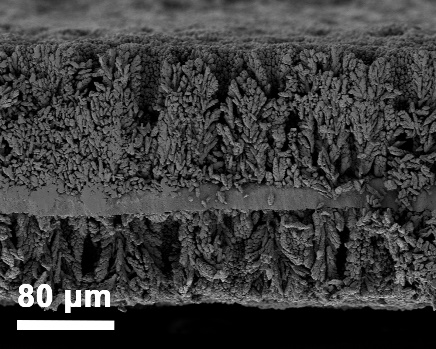


**Fig. S5** Cross-sectional SEM image of CPS-Ni electrodes

**Fig. S6** Cross-sectional SEM images with the EDS line scan of CV-Ni electrodes

**Fig. S7** XRD patterns of **a** CPS-Ni and CV-Ni electrodes, and **b** enlarged XRD image of CPS-Ni at the degree of 5 ~ 40°

**Fig. S8 a** XPS spectra and **b** the corresponding atomic content for CPS-Ni and CV-Ni electrodes

**Fig. S9** High-resolution K element XPS spectra of CPS-Ni and CV-Ni electrodes

**Fig. S10** High-resolution Ni element XPS spectra of CPS-Ni and CV-Ni electrodes

**Fig. S11 a** Setup of the *in-situ* Raman test equipment and **b** the corresponding CV curve

**Fig. S12** Attenuated total refraction Fourier transform infrared spectra (ATR-FTIR) of CV-Ni and CPS-Ni electrodes

The attenuated total refraction Fourier transform infrared spectra (ATR-FTIR) of the two electrodes were compared. As shown in the Fig. S12, the O-O vibrational frequency peak is around 1030 cm^−1^. The absorption peak intensity of O-O in CPS-Ni is increased compared with CV-Ni, which is attributed to the presence of O-O in superoxide. In addition, the red shift at 1155 cm^−1^ is caused by the stretch of superoxide [S5].

**Fig. S13** XRD pattern of CV-Ni electrodes at 0.64 V

**Fig. S14** CV curve of pure Ni substrate in KOH electrolyte containing superoxide radicals

**Fig. S15** CV curve of pure Ni substrate in KOH electrolyte without superoxide radicals

**Fig. S16** GCD curves of two Ni-substrates after each time of CV test in different electrolytes

**Fig. S17** Raman spectrum of KOH electrolyte containing superoxide radicals

**Note Figs. S14-S17**

The pure Ni electrode was prepared by the same deposition process as in the experimental part but without activation. In a 1 M KOH electrolyte containing superoxide radicals (superoxide radicals were confirmed by the peak of 1067.4 cm^−1^ in the Raman spectrum, Fig. S17), the prepared pure Ni was used as the working electrode for CV testing in a three-electrode system. The results are shown in Fig. S14. A pair of redox peaks appeared in the first cycle of CV, which was attributed to the typical conversion between Ni and Ni(OH)_2_ [S6]. In the second cycle, another pair of redox peaks appeared near 0.58/0.35 V, indicating that a new redox reaction occurred at the electrode, This is consistent with the peak of the CPS-Ni electrode and is attributed to the redox reaction between Ni and KNiO_2_.

For comparison, the pure Ni electrode in a 1 M KOH electrolyte without superoxide radicals was used for CV test, as shown in Fig. S15. The first two CV curves show only one pair of redox peaks, which is consistent with the CV curve of the CV-Ni electrode. In addition, the CV integral area of the Ni electrode containing superoxide radicals is larger than that of the Ni electrode without superoxide radicals. This is also consistent with the integral of the CV curves of the CPS-Ni and CV-Ni electrodes.

We further tested the capacity of Ni substrate in two kinds of electrolyte *via* Chronopotentiometry procedure with a current density of 1 mA cm^−2^, and the relevant GCD curves are shown in Fig. S16. The total capacity of Ni electrode containing superoxide radicals is about 0.19 mAh cm^−2^, while that of Ni electrode without superoxide radicals is about 0.07 mAh cm^−2^. These results are consistent with the capacity comparison results of CPS-Ni and CV-Ni electrodes.

In general, we have verified through the above designed experiments that the Ni substrate can be reversibly converted into KNiO_2_ under the activation of superoxide. This three-electron transfer redox reaction has a significant impact on the capacity improvement of the Ni electrode.

**Fig. S18** Raman mapping of selected region of CPS-Ni electrodes

**Fig. S19** Raman spectroscopy of CPS-Ni, CV-Ni electrodes and Ni substrate

**Fig. S20** XRD patterns of CPS-Ni electrodes after etching

**Fig. S21 a** Full spectrum and **b** high-resolution Ni element XPS spectra for CPS-Ni electrodes. **c** Relative Ni content

**Fig. S22** CV curves of **a** CPS-Ni and **b** CV-Ni electrodes at different scan rates

**Fig. S23** Electrochemical active surface area (ECSA) of different activated Ni electrodes

**Fig. S24** Enlarged view of EIS curves of **a** CPS-Ni and **b** CV-Ni electrodes

**Fig. S25** Surface SEM images of CV-Ni electrodes after 10,000 cycles at the current density of 200 mA cm^−2^

**Fig. S26** EIS curves of CPS-Ni||Zn full cell

**Fig. S27** CV curves of CPS-Ni||Zn full cell at different scan rates

**Figure S28**. **a** Digital photograph of the assembled pouch battery (electrode area = 4 × 4 cm^2^). **b** The cycling performance at 10 mA cm ^−2^

To verify the commercial viability of CPS-Ni||Zn full cells under conditions close to practical applications, we assembled a pouch battery. As shown in Fig. S28, the area capacity can be maintained at 3.13 mAh cm^−2^ after 100 cycles at a current density of 10 mA cm^−2^.

**Fig. S29** GCD curves of CPS-Ni||Zn full cell at different current densities

**Fig. S30** Bendable demonstration of the multi-wavelength photoplethysmography wearable electronics

**Supplementary References**

1. Z. Deng, H. Jiang, Y. Hu, Y. Liu, L. Zhang et al., 3D Ordered macroporous MoS_2_@C nanostructure for flexible Li-ion batteries. Adv. Mater. **29**, 1603020 (2017). <https://doi.org/10.1002/adma.201603020>
2. Q. Chen, J. Li, C. Liao, G. Hu, Y. Fu, O. K. Asare et al., Ni foam supported NiO nanosheets as high-performance free-standing electrodes for hybrid supercapacitors and Ni–Zn batteries. J. Mater. Chem. A **6**, 19488-19494 (2018). <https://doi.org/10.1039/C8TA07574C>
3. J. Ma, S. Zheng, L. Chi, Y. Liu, Y. Zhang et al., 3D printing flexible sodium‐ion microbatteries with ultrahigh areal capacity and robust rate capability. Adv. Mater. **34**, 2205569 (2022). <https://doi.org/10.1002/adma.202205569>
4. X. Liu, Y. Liu, Q. Zhang, S. Zhou, X. Li et al., Integrating aperiodic 3D porous electrodes into 3D batteries through spray‐deposited polymer electrolytes. Adv. Energy Mater. 2401330 (2024). <https://doi.org/10.1002/aenm.202401330>
5. M. Hayyan, M. Hashim, I. AlNashef, Superoxide ion: generation and chemical implications. Chemi. Rev. **116**, 3029-3085 (2016). https://doi.org/10.1021/acs.chemrev.5b00407
6. Y. Duan, G. You, Z. Zhu, L. Lv, X. Liao et al., Reconstructed NiCo alloy enables high-rate Ni-Zn microbattery with high capacity. Coatings **13**, 603 (2023). <https://doi.org/10.3390/coatings13030603>
